# Cellular prion protein controls stem cell-like properties of human glioblastoma tumor-initiating cells

**DOI:** 10.18632/oncotarget.9575

**Published:** 2016-05-24

**Authors:** Alessandro Corsaro, Adriana Bajetto, Stefano Thellung, Giulia Begani, Valentina Villa, Mario Nizzari, Alessandra Pattarozzi, Agnese Solari, Monica Gatti, Aldo Pagano, Roberto Würth, Antonio Daga, Federica Barbieri, Tullio Florio

**Affiliations:** ^1^ Sezione di Farmacologia, Dipartimento di Medicina Interna, University of Genova, Genova, Italy; ^2^ Centro di Eccellenza per la Ricerca Biomedica (CEBR), University of Genova, Genova, Italy; ^3^ Department of Experimental Medicine, University of Genova, Genova, Italy; ^4^ IRCCS AOU San Martino - IST, Genova, Italy

**Keywords:** prion protein, glioblastoma, cancer stem cells, in vivo tumorigenicity, GFAP

## Abstract

Prion protein (PrP^C^) is a cell surface glycoprotein whose misfolding is responsible for prion diseases. Although its physiological role is not completely defined, several lines of evidence propose that PrP^C^ is involved in self-renewal, pluripotency gene expression, proliferation and differentiation of neural stem cells. Moreover, PrP^C^ regulates different biological functions in human tumors, including glioblastoma (GBM). We analyzed the role of PrP^C^ in GBM cell pathogenicity focusing on tumor-initiating cells (TICs, or cancer stem cells, CSCs), the subpopulation responsible for development, progression and recurrence of most malignancies. Analyzing four GBM CSC-enriched cultures, we show that PrP^C^ expression is directly correlated with the proliferation rate of the cells. To better define its role in CSC biology, we knocked-down PrP^C^ expression in two of these GBM-derived CSC cultures by specific lentiviral-delivered shRNAs. We provide evidence that CSC proliferation rate, spherogenesis and *in vivo* tumorigenicity are significantly inhibited in PrP^C^ down-regulated cells. Moreover, PrP^C^ down-regulation caused loss of expression of the stemness and self-renewal markers (NANOG, Sox2) and the activation of differentiation pathways (*i.e.* increased GFAP expression). Our results suggest that PrP^C^ controls the stemness properties of human GBM CSCs and that its down-regulation induces the acquisition of a more differentiated and less oncogenic phenotype.

## INTRODUCTION

Cellular prion protein (PrP^C^) is a cell surface glycoprotein, highly conserved in all mammalian species. PrP^C^ is considered at the basis of the pathogenesis of prion diseases, in which the fundamental event is its misfolding into a protease-insensitive, amyloidogenic isoform (PrP^Sc^) [1]. Misfolded PrP^C^ accumulates in intra- and extracellular deposits as insoluble protein aggregates [2, 3] responsible of neurotoxicity and astrogliosis [4–8]. The conversion of PrP^C^ into PrP^Sc^ consists in a radical modification of its three-dimensional structure and, consequently, of its biochemical and biological properties [9–11]. The “protein only” hypothesis [1] postulates that PrP^Sc^, in an autocatalytic reaction, duplicates itself inducing its abnormal spatial structure on newly synthesized PrP^C^ molecules [12, 13]. PrP^C^ is highly expressed within the central nervous system (CNS), although its content varies among distinct brain regions, among different cell types and/or neurons with distinct neurochemical phenotypes [1]. Various cellular components of the immune system, in bone marrow, blood, and peripheral tissues, also express substantial amounts of PrP^C^ [14]. However, even though several studies have been performed to define its physiological function, there are still no unequivocal data able to define a precise cellular function of PrP^C^.

*PRNP* (the PrP^C^ gene)-knockout experiments did not evidence particular alterations in mice, indicating that PrP^C^ is not essential for normal development or that PrP^C^ loss of function can be compensated by other molecules [15]. In search for a physiological function for this protein, PrP^C^ was proposed to protect neurons against cell death and oxidative stress [16], to control copper metabolism [17], to regulate cell cycle [18], synaptic transmission [19], and cell adhesion [20], and to activate the immune system [21]. Interestingly, more recent studies suggested that PrP^C^ plays a role in pluripotency and differentiation of embryonic stem cells [22], cell proliferation and differentiation [23–28], and muscle cell regeneration [29], through the direct activation of the Src-family kinase Fyn, at least as far as the CNS effects [30]. Starting from these observations PrP^C^ has been intriguingly involved in the development of human tumors [22, 31], including glioblastoma [32, 33], and gastric [34], breast [35], prostate [36], and colorectal [37] carcinomas. For example, PrP^C^ expression was correlated with increased cell proliferation in gastric cancer cell lines [18, 38], and PrP^C^ overexpression was shown to provide cancer cells with resistance to cytotoxic agents [36], and higher invasive properties [39].

Cancer stem cells (CSCs, also called tumor-initiating cells, TICs, due to their *in vivo* tumorigenic activity) derive their denomination from several phenotypical and functional characteristics shared with normal stem cells [40] and were identified over a decade ago in glioblastoma (GBM), the most common and aggressive CNS tumor [41]. GBM CSCs are resistant to conventional chemo-radiotherapy due to high activity of DNA repairing enzyme and drug efflux pumps, and their persistence after cytotoxic therapy is believed to determine tumor recurrence [42, 43]. In virtue of these proprieties, GBM CSCs represent the focus for novel targeted therapies [44, 45]; moreover, the identification of specific signaling pathways responsible for the retention of stemness, might have a significant translational relevance, contributing to the eradication of this cell subpopulation.

CSC-enriched cultures can be obtained from post-surgical GBM specimens using the protocols adopted to isolate neural stem cells [46]. They are able to grow indefinitely in serum-free medium, supplemented with growth factors (EGF and bFGF) [47], as non-adherent cultures that generate three-dimensional spheroids, an *in vitro* index of self-renewal [48]; moreover, CSC cultures can differentiate into different brain cell lineages and are tumorigenic when orthotopically xenografted in immunodeficient mice [49].

Here we report the role of PrP^C^ in regulating CSCs phenotype and functioning. In particular, we analyzed the effects of the down-regulation of PrP^C^ expression in CSCs isolated from human GBMs. We report that PrP^C^ expression restrains GBM CSCs from differentiation, conferring them distinctive stem cell-like features, such as self-renewal ability and *in vivo* tumorigenicity.

## RESULTS

### PrP^C^ expression level correlates with the proliferation rate of human GBM CSCs

**Figure 1:**
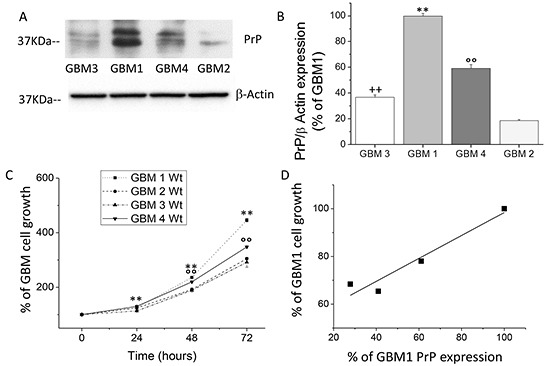
**A.** Representative immunoblot analysis of PrP^C^ protein level in 4 different wt GBM CSC cultures. PrP^C^ content was determined by 3F4 immunoreactivity. Immunoblotting for β-actin was used to normalize the results for the total content of proteins. **B.** Quantification of PrP^C^ protein level, reported as densitometric analysis of blots as in panel A, derived from three independent experiments and expressed as percentage of wt GBM2, 3, 4 immunoreactive bands *vs.* wt GBM1. **p < 0.01 *vs.* wt GBM2, 3, 4 CSCs, °°p < 0.01 *vs.* wt GBM 1, 2, 3 wt CSCs; ^++^p < 0.01 *vs.* wt GBM 1, 2, 4 CSCs. **C.** Growth curve of wt GBM 1-4 CSC cultures. Cell proliferation was evaluated by MTT reduction test. Values, taken as percentage of the values at time 0, are the average of two independent experiments, performed in quadruplicate. **p < 0.01 *vs.* GBM2,3,4, wt cells and °°p < 0.01 *vs.* GBM1,2,3 wt cells **D.** Linear regression analysis of the relationship between PrP^C^ expression and the proliferation rate of GBM1-4 CSC cultures.

To establish a functional role for PrP^C^ in human GBM CSCs, we analyzed the relationship between native PrP^C^ expression levels and proliferation rate in four different CSC-enriched cultures, named GBM1-4, isolated from human GBMs. PrP^C^ expression was assessed by immunoblot (Figures 1A and 1B). We observed significant differences in PrP^C^ expression among CSCs from the different tumors. Densitometric analysis of immunoreactive bands demonstrated that GBM1 CSCs express the highest level of PrP^C^ respect to the other cultures, being four times the expression observed in GBM2, two times that of GBM3, about 1 time more than GBM4 (Figure 1B). By MTT reduction assay, we analyzed, up to 72 hrs., the CSC proliferation rate. As shown in Figure 1C, GBM1 CSCs displayed the highest proliferation rate, followed by GBM4, while GBM3 and GBM2 CSCs have slower duplication time. Linear regression analysis, correlating PrP^C^ expression and cell proliferation at 72 hrs., revealed a direct correlation between these parameters (Figure 1D), with a highly significant statistical relationship (R^2^: 0.9).

### Stable silencing of PrP^C^ mRNA and protein in human GBM stem cell-enriched cultures by shRNA

**Figure 2:**
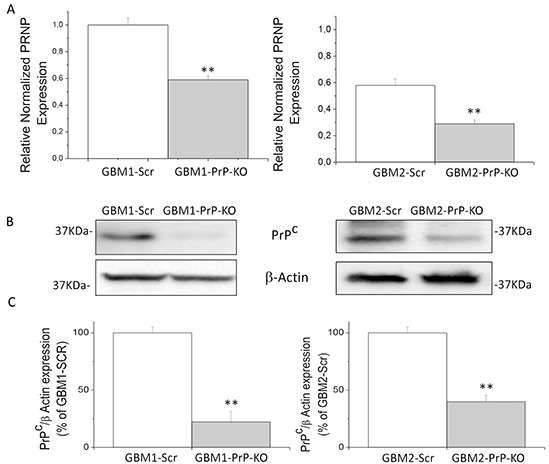
**A.** Prion protein mRNA expression in GBM1 (left) and GBM2 (right), evaluated by quantitative RT-PCR. *PRNP* expression values in GBM PrP-KO cells (grey bars) were normalized *vs.* respective GBM-Scr cells (white bars). Results are expressed as means ± SEM. **p < 0.01 *vs.* respective GBM-Scr cells. **B.** Immunoblotting analysis of PrP^C^ protein level in GBM1 (left) and GBM2 (right) cells. PrP^C^ content was determined by 3F4 immunoreactivity by Western blot. Immunoblotting for β-actin was used to normalize the results for the total content of proteins. **C.** Quantification of PrP^C^ protein level, reported as densitometric analysis of the blots derived from three independent silencing experiments and as expressed percentage of GBM PrP-KO (grey bars) 3F4-immunoreactive bands *vs.* GBM-Scr (white bars). **p < 0.01 *vs.* respective GBM-Scr cells.

To delve deeper in to the role of PrP^C^ expression in GBM cell proliferation, and analyze the role of this protein in the defining properties of CSCs, we evaluated the effects of PrP^C^ downregulation by gene silencing using specific shRNA. We analyzed CSCs from GBM1 and GBM2, representing the cultures with the highest and the lowest levels of PrP^C^. GBM1 and GBM2 CSCs were transfected with PrP shRNA, using a pool of 3 target-specific lentiviral plasmids, each encoding 19-25 nt (plus hairpin) designed to knock-down gene expression. To generate control cultures, GBM1 and GBM2 cells were also transfected with a plasmid encoding a scrambled (Scr) shRNA sequence, which does not target any known cellular mRNA. Transfected cells were selected with puromycin and the stable PrP^C^ knock-down cultures were named GBM1-PrP-KO and GBM2-PrP-KO, while cells transfected with the scrambled shRNA sequence were named GBM1-Scr and GBM2-Scr. The efficiency of PrP^C^ knock-down was verified by quantitative Real Time PCR (qRT-PCR) analysis (Figure 2A) and immunoblot (Figures 2B and 2C), showing a significant reduction of PrP^C^ expression in both GBM-PrP-KO cell cultures. In particular, densitometric analysis of immunoreactive bands showed a decrease of PrP^C^ protein expression of about 80% in GBM1-PrP-KO and 60% for GBM2-PrP-KO cells *vs.* respective controls (GBM-Scr) (Figure 2C).

### PrP^C^ knockdown reduces proliferation of GBM CSCs

Proliferation of GBM PrP-KO and Scr cells was tested by MTT reduction assay, that measures mitochondrial activity as an index of cell viability being proportional to cell number, by counting cell number, using an automated cell counter, and by BrdU incorporation assay, which evaluates DNA synthesis.

**Figure 3:**
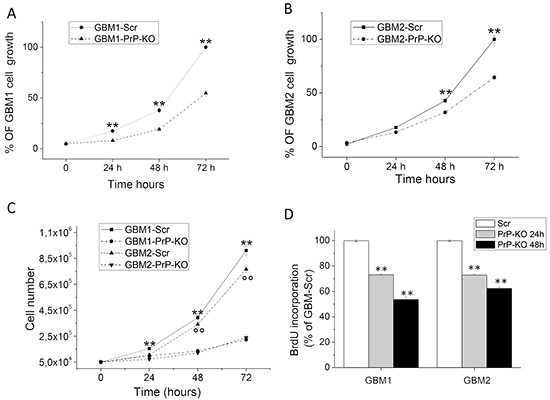
**A-B.** Proliferation curves of GBM1-PrP-KO and GBM1-Scr (A) and GBM2-PrP-KO and GBM2-Scr (B) cells assessed by MTT assay after 24, 48 and 72 hours. Each point represents the average of three experiments performed in quadruplicate. **p < 0.01 *vs.* respective GBM-PrP-KO cells. **C.** Proliferation curves of GBM1-PrP-KO, GBM1-Scr, GBM2-PrP-KO and GBM2-Scr cells assessed by cell count after 24, 48 and 72 hours of proliferation. Each point represents the average of three experiments performed in quadruplicate. **p < 0.01 *vs.* GBM1-PrP-KO. °°p < 0.01 *vs.* GBM2-PrP-KO. **D.** DNA synthesis rate of GBM1-PrP-KO and GBM2-PrP-KO cells in comparison with respective GBM-Scr control cells, determined by BrdU incorporation assay after 24 and 48 hours. Data are expressed as a percentage of respective GBM-Scr cells, and each point represents the average of three experiments, performed in quadruplicate. **p < 0.01 *vs.* respective GBM-Scr cells.

For MTT assays, GBM CSCs were plated at the concentration of 2.5×10^4^/well, and cell number was evaluated after 24, 48 and 72 hours. GBM1- and GBM2-PrP-KO cells showed a significantly reduced proliferation rate (Figures 3A and 3B), as compared to the respective GBM-Scr control cells. The effect was more evident in GBM1-PrP-KO in which PrP^C^ mRNA silencing was more pronounced, but in both cell cultures the differences become bigger with the increasing of the time.

It is important underline that, in MTT experiments, we did not observe significant difference in growth curve between non-transfected (*wild type*) GBM CSCs and GBM-Scr (control transfected cells, data not shown), confirming that the expression of the Scr sequence does not influence the proliferation rate of GBM CSCs. These experiments were confirmed by automated cell counting (Figure 3C) evaluated up to 3 days after cell plating. GBM1 Scr cells showed the highest proliferation levels and the two PrP-KO cultures the slowest duplication activity. Moreover, the quantification of cell death by Trypan Blue exclusion test, did not reveal significant difference between GBM-Scr and GBM-PrP-KO cell cultures (about 5% in all the cultures evaluated, data not shown) suggesting that the reduction of PrP expression is not cause of toxicity but effectively interferes with cell proliferation. These data were confirmed in experiments in which BrdU incorporation, during DNA synthesis, was measured by specific ELISA (Figure 3D). GBM cells were plated at the concentration of 1×10^4^/well, and BrdU incorporation evaluated after 24 and 48 hrs. DNA synthesis in GBM1-PrP-KO and GBM2-PrP-KO cells was significantly reduced as compared to the respective GBM Scr cells (about −30% after 24 hours in both GBM-PrP-KO cultures, about −50% in GBM1-PrP-KO and about −40% in GBM2-PrP-KO cells, after 48 hours).

Finally, the role of PrP^C^ in GBM cell proliferation was further confirmed in *wild type* GBM CSCs, analyzing the effect of two anti-PrP antibodies in the MTT assay. In fact, it was reported that the anti-PrP antibodies causes the inhibition of PrP^C^ dimerization and the consequent intracellular signaling, inducing neurotoxic effects in neural progenitors [50, 51] and reduced tumorigenicity of colon CSCs [37]. We tested the effect of two specific anti-PrP antibodies, the clones 3F4 (epitope corresponding to amino acids 109–112 in the human PrP) and SAF 32 (epitope corresponding to amino acids 63-94 in the human PrP) on *wild type* GBM1 and GBM2 CSC proliferation. The treatment for 48 hrs. with different antibody concentrations confirmed that the interference with PrP^C^ signaling causes a statistically significant reduction of the proliferation rate (−28 and −22%, *p*<0.01, with 1mg/ml of both 3F4 and SAF32, respectively in GBM1 cells, and −19%, *p*<0.01 and −13%, p<0.05 with 2mg/ml of 3F4 and SAF32, respectively in GBM2 cells, data not shown). These findings confirm that PrP^C^ plays an important role in the regulation of GBM CSC proliferation, and that the effects observed in the PrP^C^-KO cells are effectively dependent on the reduction of the PrP^C^ signaling and not due to shRNA off-target effects.

### PrP^C^ controls clonogenicity and self-renewal of GBM CSCs

**Figure 4:**
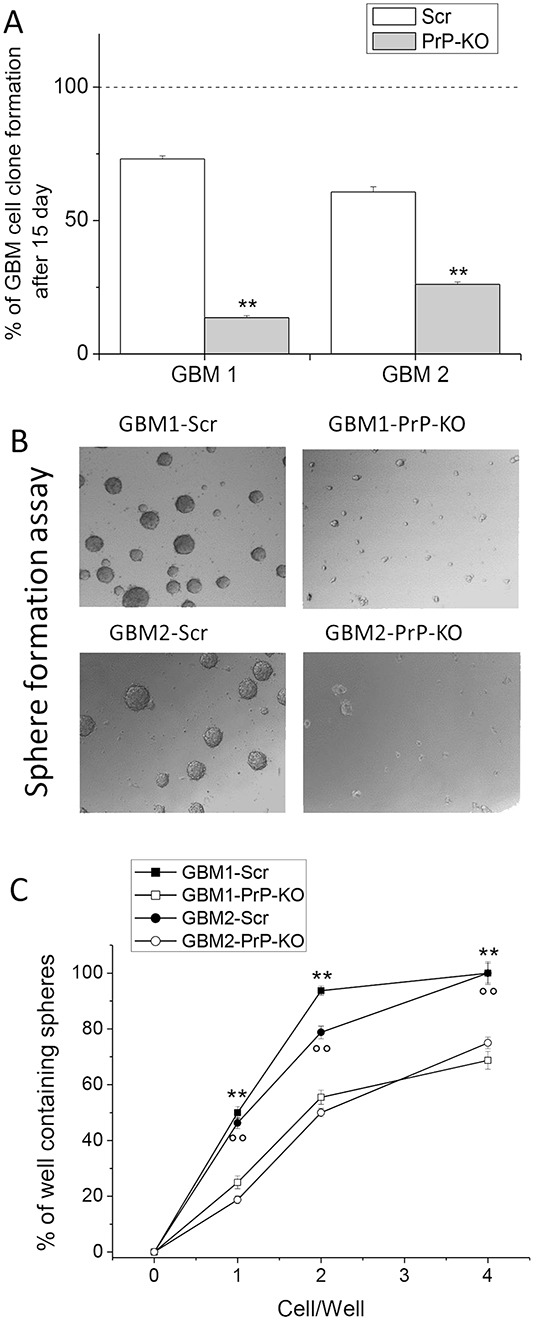
**A.** Quantification of clonogenic activity of GBM1- and GBM2- Scr and PrP-KO cells, expressed as percentage of the number of plated cells, assumed as 100%. Each point represents the average of three experiments performed in quadruplicate. **p < 0.01 *vs.* GBM-Scr cells. **B.** Representative micrographs of the sphere formed by GBM-Scr and GBM-PrP-KO cells after 7 days of growth (using 5x/012 Leica Objective). These data underline that PrP-KO cells derived from both GBM1 and GBM2 form rare small spheroids. **C.** Limiting dilution analysis comparing stem cell of sphere formation between by GBM-Scr and GBM-PrP-KO cells after 15 days of growth in complete medium in 96 well ultra-low adherent plates. Plating density ranges from 100 to 0.01 cells/well. **p<0.01 *vs.* GBM1-PRP-KO cells, °°p<0.01 *vs.* GBM2-PRP-KO cells.

To verify whether, besides proliferation, PrP^C^ down-regulation also affects distinctive features of GBM CSCs, we compared the clonogenic activity of GBM-Scr and GBM-KO cultures, as *in vitro* index of stemness. GBM-Scr and PrP-KO cells were seeded onto matrigel, by limiting dilution in order to obtain a concentration lower than 1 cell/well. After 15 days in culture, individual wells were visually inspected under a phase contrast microscope and the developed clones counted. A dramatic reduction in clone formation occurred in both GBM-PrP-KO cultures as compared to the respective GBM-Scr cells (Figure 4A). Again, the reduction in clonogenic activity was much more evident in PrP-silenced CSCs derived from GBM1 than in cells isolated from GBM2, providing a further correlation between the levels of PrP^C^ down-regulation and CSC functional properties.

The loss of clonogenicity in GBM-PrP-KO cells suggested that other key features of CSC subpopulation might be affected by the down-regulation of PrP^C^ expression. Thus, we analyzed the differential ability of PrP-KO and Scr cells to grow as gliomaspheres, considered an *in vitro* index of self-renewal activity [52]. GBM-Scr and PrP-KO cells were seeded at the concentration of 1000 cells/well, without matrigel coating. After 15 days, sphere formation was analyzed by light microscopy. Contrarily to control GBM-Scr, GBM-PrP-KO cells are unable to form spheroids, resulting in the formation of only few small cell aggregates (Figure 4B). Moreover, we performed sphere limiting dilution experiments. As reported in Figure 4C, a dramatic reduction in the percentage of sphere formation occurred in both GBM-PrP-KO cultures compared to the respective GBM-Scr cells. Counting the number of wells in which spheres developed, we observed that the difference in sphere formation efficiency was maximal in the wells containing only one cell, being detected a sphere in less than 20% of the wells containing PrPC-KO CSC, *vs*. about 50% of the GBM-Scr. The estimation of sphere-forming probability by linear regression analysis as described by [53], showed that the down-regulation of PrPC reduced the spherogenic activity by more than three times in GBM1 and by two-fold in GBM2. These data clearly support the possibility that the lack of PrP^C^ expression in GBM CSCs affects not only *in vitro* proliferation rate but also relevant stemness features, such as self-renewal.

### PrP^C^ expression controls the stem cell-like phenotype in human GBM CSCs

To confirm the role of PrP^C^ as a stemness regulator of GBM CSCs, we investigated phenotype alterations induced by PrP^C^ down-regulation, focusing on mRNA and protein levels of Sox2 and NANOG, two transcription factors essential for maintaining self-renewal and pluripotency, not only in human embryonic [54] and neural stem cells [55] but also in human GBM CSCs [56].

**Figure 5:**
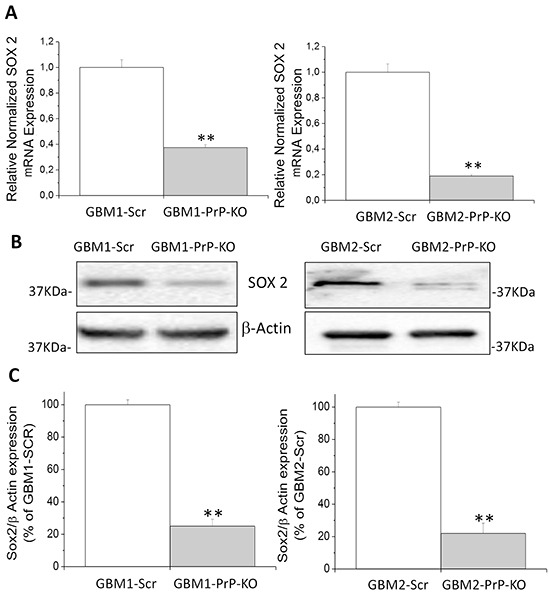
**A.** Expression of Sox2 mRNA in GBM1- and GBM2-PrP-KO cells, evaluated by quantitative RT-PCR. Sox2 expression values were normalized to respective GBM-Scr cells (white bars). Results are expressed as means ± SEM. **p<0.01 *vs.* respective GBM-Scr cells. **B.** Representative immunoblots of Sox2 protein expression level in GBM-Scr and GBM PrP-KO cells. Immunoblot analysis of β-actin levels was performed to normalize the data for the total protein input of cell lysates. **C.** Densitometric analysis of Sox2 expression from three independent Western blot experiments and expressed as percentage of GBM-Scr cells ± SEM. **p<0.01 *vs.* respective GBM-Scr cells.

Sox2 mRNA content, analyzed by qRT-PCR, was significantly reduced in both GBM-PrP-KO cells (about −70% in GBM1- and GBM2-derived CSCs) (Figure 5A). These results were confirmed at protein level by Western blot (Figures 5B and 5C), in which GBM1- and GBM2-PrP-KO displayed a significant reduction of Sox2 expression (approximately −80% of the respective GBM-Scr cells).

**Figure 6:**
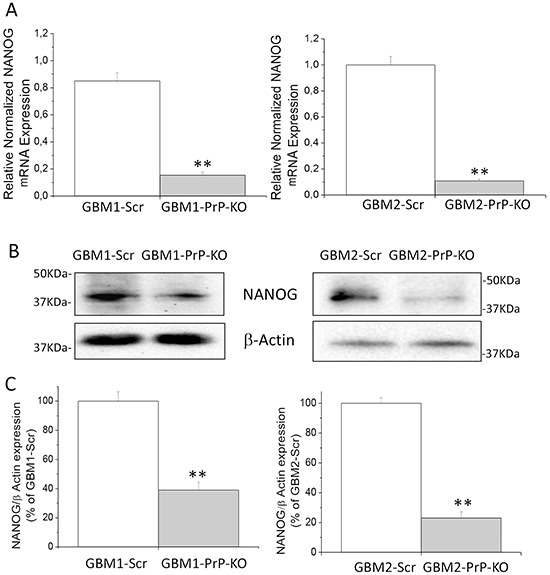
**A.** Expression of NANOG mRNA in GBM1 and GBM2-Scr and PrP-KO cells, evaluated by quantitative RT-PCR. NANOG expression values were normalized to respective GBM-Scr cells (white bars). Results are expressed as means ± SEM. **p < 0.01 *vs.* respective GBM-Scr cells. **B.** Representative immunoblots of NANOG protein expression level in GBM-Scr and GBM PrP-KO cells. Immunoblot analysis of β-actin levels was performed to normalize the data for the total protein input of cell lysates. **C.** Densitometric analysis of NANOG expression from three independent Western blot experiments and expressed as percentage of GBM-Scr cells ± SEM.**p<0.01 *vs.* respective GBM-Scr cells.

Similar results were obtained as far as the expression of NANOG, a transcription factor expressed in CSCs in which regulates cell death and proliferation [57, 58]. By qRT-PCR and Western blot experiments, we demonstrated that NANOG mRNA and protein contents (Figure 6A, 6B and 6C) are significantly reduced in GBM CSC cultures in which PrP^C^ is down-regulated.

**Figure 7:**
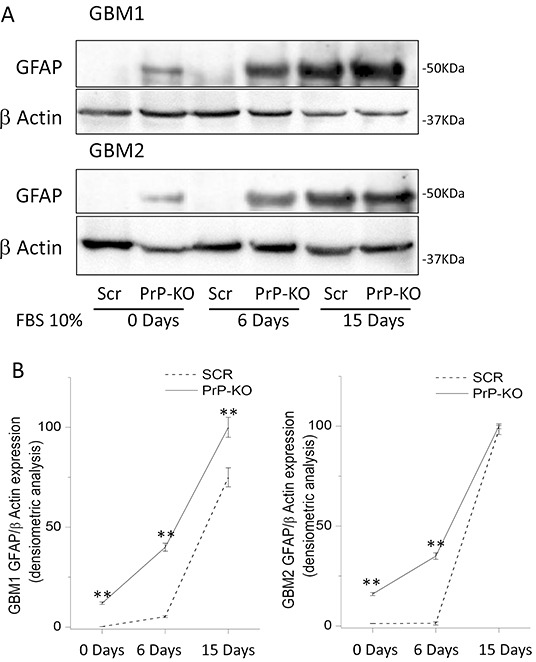
**A.** Representative immunoblots of GFAP expression in GBM-Scr and GBM-PrP-KO cells. Cells were analyzed after growing in stem cell permissive medium (time 0), or after cell differentiation, induced by incubation for 6 or 15 days in medium deprived of growth factors and additioned with 10% FBS. Immunoblot analysis of β-actin was performed to verify the total protein input in the cell lysates. **B.** GFAP expression is reported as densitometric analysis of the gels derived from three independent experiments and expressed as percentage of the highest intensity value of GFAP/β-actin ratio. Cells were analyzed after growing the cells in stem cell permissive medium (day 0), or after inducing cell differentiation by incubation for 6 or 15 days in medium deprived of growth factors and additioned with 10% FBS. **p<0.01 *vs.* respective GBM-Scr cells.

**Figure 8:**
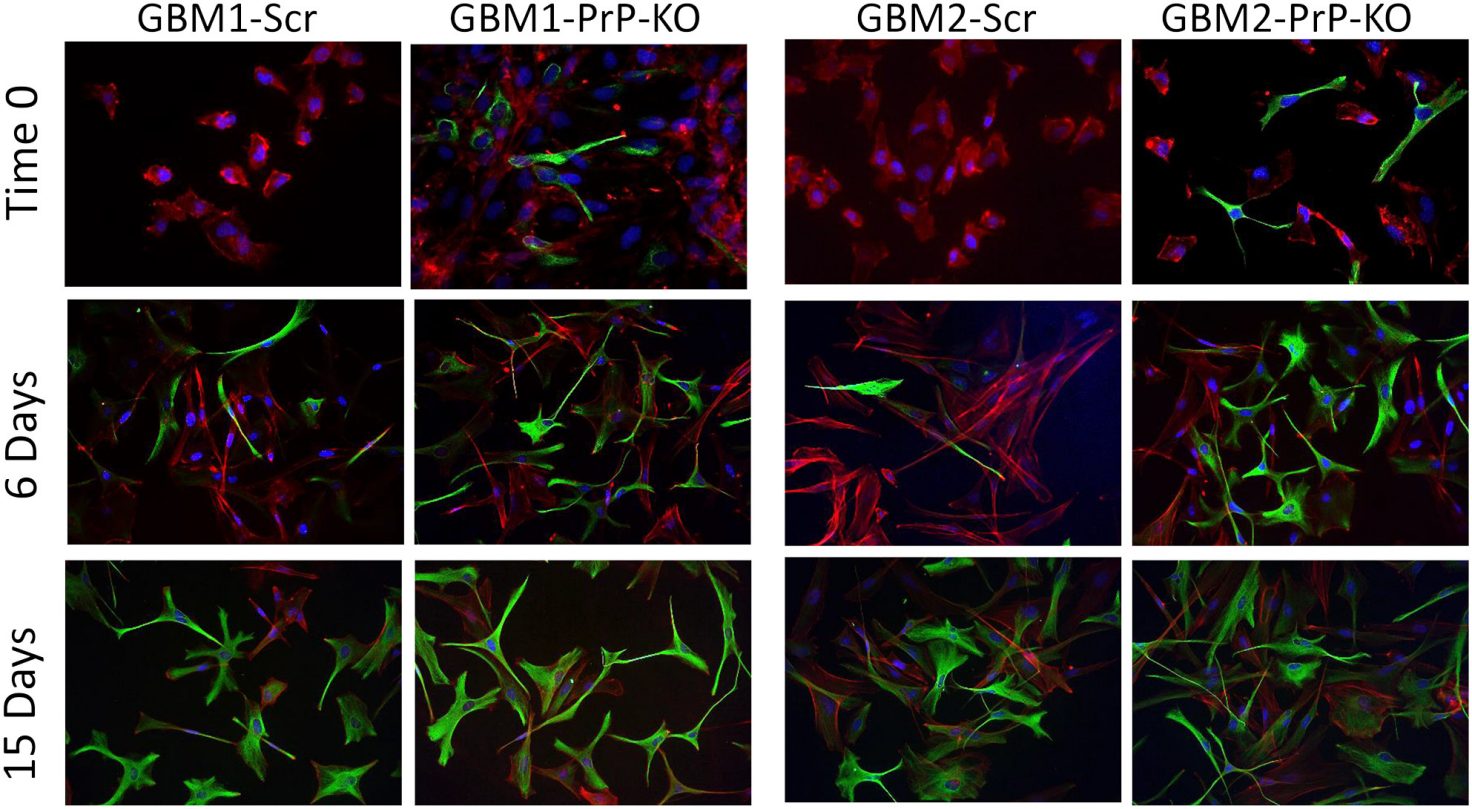
Immunofluorescence analysis of GFAP expression (green) at time 0, after 6 and 15 days of differentiation in medium containing 10% FBS Nuclei were counterstained with DAPI (blue) and cells morphology was evidenced by staining with Dil, a lipophilic red-fluorescent dye. Magnification 40X.

In GBM-PrP-KO CSCs the loss of stemness regulators was associated with increased levels of the astrocytic differentiation marker glial fibrillary acidic protein (GFAP). We show that, while GBM-Scr cells, grown in serum-free, EGF/bFGF-containing medium, do not show signs of spontaneous differentiation (*i.e.* no GFAP expression evaluated by both Western blot and immunocytofluorescence), GBM-PrP-KO cultures (from both GBM1 and GBM2), grown in the same conditions, display a spontaneous basal expression of GFAP in several cells (Figures 7 and 8). GBM CSCs can be induced to differentiate by shifting the culture conditions from a growth factor-containing medium to a medium enriched with 10% FBS [46, 49]. Similarly to native GBM1 and GBM2 CSCs (data not shown), GBM1- and GBM2-Scr, after 2 weeks of growth in differentiating culture conditions (10% FBS) underwent to morphological changes and increased GFAP expression in almost all the cells (Figure 7 and 8). However, this effect was greatly accelerated in GBM1-PrP-KO and GBM2-PrP-KO cells in which a significant increase in GFAP expression was evident after only 6 days from the beginning of the differentiation process (evaluated by both Western blot and immunocytofluorescence, Figures 7 and 8).

These data, altogether with the reduced expression of transcription factors related to pluripotency and self-renewal, clearly demonstrated that PrP^C^ expression controls the subtle equilibrium between stemness and differentiation of human GBM CSCs.

### PrP^C^ expression influences tube formation in human GBM CSCs

Normal neural stem cells are able to differentiate into functional endothelial cells. The connection between neural stem cells and the endothelial compartment seems to be critical in glioblastoma, where CSCs closely interact with the vascular niche and promote angiogenesis through the release of vascular endothelial growth factor and CXCL12 in an autocrine/paracrine manner [59–62]

**Figure 9:**
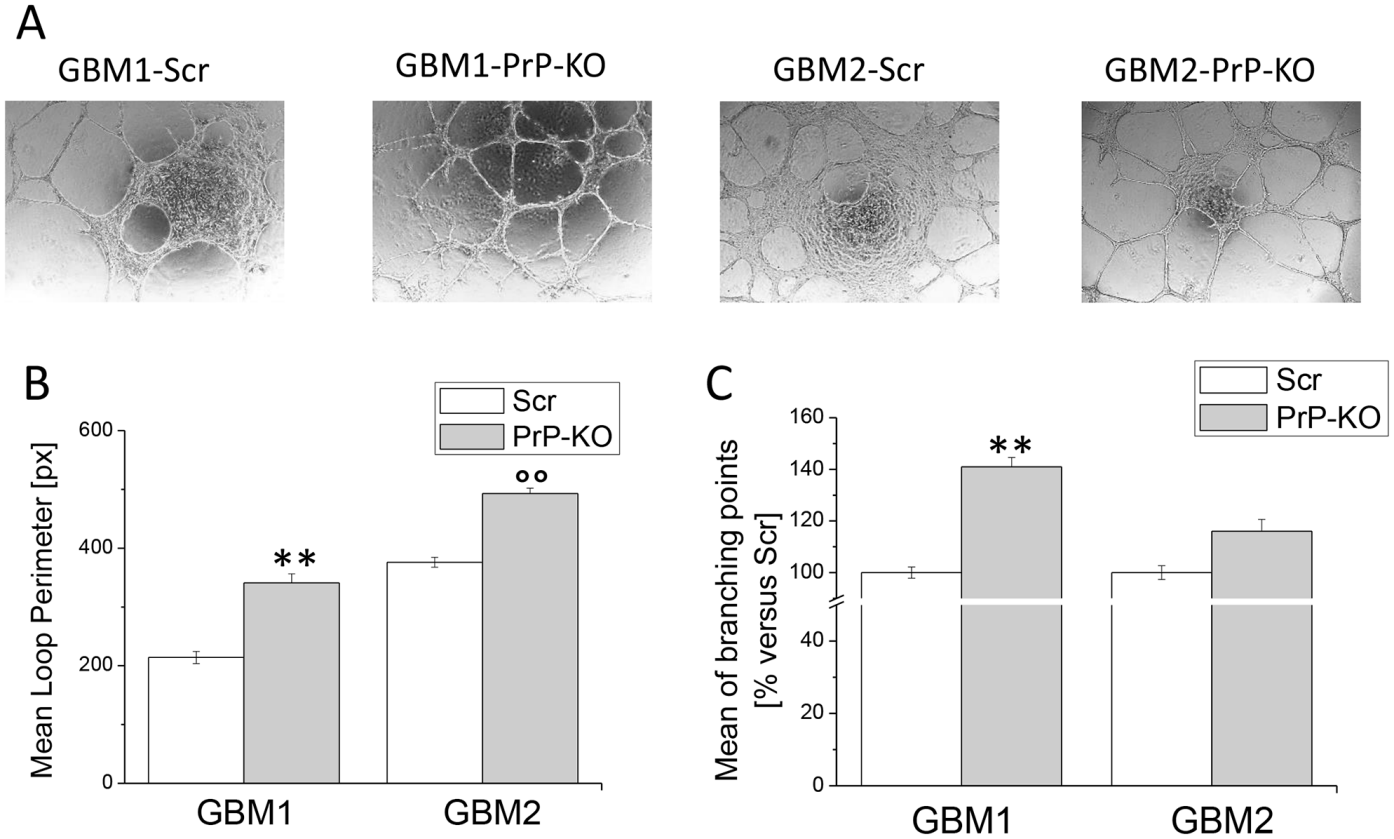
Effect of PrP^C^ down-regulation on the ability of GBM CSCs to form endothelial tubes **A.** Representative micrographs of tube formation in GBM-Scr and GBM-PrP-KO cells after 24h of growth in endothelial differentiation medium. **B.** Mean loops tube perimeter and **C.** mean branching points formation, induced in GBM cells by endothelial transdifferentiation. Perimeters are expressed in pixel. Data are expressed as percentage of respective GBM-Scr cell value. Each point represents the average of three independent experiments. **p<0.01 *vs.* GBM1-Scr cells, °°p<0.01 *vs.* GBM2-Scr cells

Starting from this evidence, we evaluated whether PrP^C^ expression influences GBM-CSCs ability to transdifferentiate into endothelial-like cells performing an *in vitro* tube formation assay. In order to induce tube formation, CSCs were seeded on matrigel-coated “μ–Slide Angiogenesis” and incubated at 37°C for 24h, in medium formulated to sustain endothelial cell growth *in vitro*. As shown in Figure 9A, in these experimental conditions both GBM-PrP-KO cultures were able to form more vessel-like structures than GBM-PrP Scr. This process was quantified by mean loop perimeter (Figure 9B) and mean of branching points (Figure 9C), confirming that PrP^C^ down-regulation favors GBM-CSC differentiation process.

### PrP knockdown reduces tumorigenic potential of GBM CSCs

**Figure 10:**
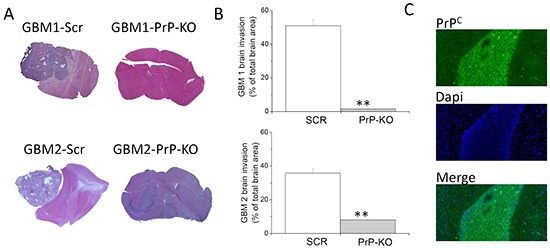
Role of PrP^C^ in the *in vivo* tumorigenicity of human GBM CSCs **A.** Representative images of tumors developed in NOD/SCID mice orthotopically grafted with GBM1- or GBM2-Scr, and GBM1- or GBM2-PrP-KO cells. After sacrifice of the animals, brains were fixed and stained with hematoxylin and eosin (H&E), showing that GBM1- and GBM2-Scr cells developed larger and more invasive tumors, as compared to the respective GBM PrP-KO cells. **B.** Quantification of GBM 1 and 2 brain invasion area, evaluated as percentage of total brain area. **p<0.01 *vs.* respective GBM-Scr cells. **C.** Representative immunofluorescence analysis of PrP expression (antibody 3F4, green) in tumors originated from GBM1-PrP-KO. Nuclei were counterstained with DAPI (blue).

According to the hierarchical tumorigenesis theory, only the small CSC subpopulation, among all the cells in the tumor mass, is endowed with tumorigenic potential (thus they are called tumor-initiating cells, TIC). To demonstrate that PrP^C^ is involved in this fundamental CSC property, we compared the ability of GBM-Scr and GBM-PrP-KO cells to develop tumors in an orthotopic mouse model. Cells (10,000/mice) were intracranially xenografted in NOD/SCID mice [46, 49]. Tumors were allowed to develop for about 3 months and animal receiving control GBM-Scr cells were sacrificed when showing signs of suffering due to the excessive growth of the tumor. Mice injected with GBM-PrP-KO cells, that did not display pathological sings during the observation period, were sacrificed along the last control mice. By immunohistochemical analysis we observed that all the mice studied developed tumors, but brains xenografted with both GBM1- and GBM2-Scr cells showed the presence of large tumor masses, with a diffuse tumor invasion of the brain parenchyma (Figure 10A, 10B), while the injection of GBM1- and GBM2-PrP-KO cells, analyzed at the same time after the injection, caused the formation of very small tumors. Interestingly, tumors originated from GBM1-PrP-KO showed a significant lower dimension than those formed by GBM2-PrP-KO, further confirming a relationship between the levels of PrP^C^ expression and the loss of CSC features. Moreover, cells composing these minimal tumors showed expression of PrP^C^, when labeled with 3F4 anti-human PrP^C^ antibody (Figure 10C). This observation suggests that these tumors originate from small cell subpopulations, which, once injected in vivo (or possibly already present in very small percentage *in vitro*) lose the mRNA silencer, and the re-expression of PrP^C^ granted the reacquisition of the *in vivo* tumorigenic activity.

All together these results clearly confirm that PrP^C^ is an important requirement for CSC to develop GBM *in vivo*.

## DISCUSSION

In the past years, one of the main issue in prion research has been the quest for a physiological role for PrP^C^ [63]. In fact, although highly concentrated within the CNS, this membrane-bound extracellular glycoprotein, is ubiquitous in humans and extremely conserved throughout the evolution. In this context, several studies proposed that PrP^C^ is involved in the regulation of stem cell self-renewal, proliferation and differentiation [64, 65]. Moreover, more recently it was suggested that PrP^C^ might also promote human tumor development and diffusion, and the induction of drug resistance [22, 36]. PrP^C^ overexpression was observed in gastric cancer lesions as compared to non-tumor tissues representing a predictive marker of poor prognosis [66], while PrP^C^ down-regulation reduced tumor cell proliferation [67] or caused cell death *via* the activation of the autophagic pathway [68]. However, most of these studies have been performed in continuous cell lines evaluating the effects of PrP^C^ on the biological behavior of the cells. According to the current theory of tumorigenesis, CSCs are pivotal mediators of tumor development, representing the real pharmacological target to obtain efficacious treatments [43, 69]. Conversely, it was shown that prolonged *in vitro* growth in FBS-containing medium (as occurs in established cell lines) causes the loss of the main malignant features observed in CSCs (*in vivo* tumorigenesis, self-renewal, etc.) altering the pharmacological sensitivity of these cultures [70]. As far as PrP^C^ is concerned, only few studies directly analyzed its role in the regulation of CSC biology. It was shown that PrP^C^ silencing reduced the metastatic potential of CSCs sorted from human colorectal cancers as CD44^+^, a stem-like membrane receptor involved in cell adhesion, motility, and metastasis. This effect was mimicked by the administration of anti-PrP antibodies [37]. Moreover, the direct interaction between PrP^C^ and CD44 is determinant for the multi-drug resistant phenotype in breast cancer cells [67].

In the current study, we examined the role of PrP^C^ in GBM stem cell functioning. Starting from the observation of a direct correlation between PrP^C^ expression levels and proliferation rate of four CSC-enriched cultures derived from individual human GBMs, we deeper analyzed this correlation taking advantage of two GBM CSC cultures in which PrP^C^ expression was significantly reduced by the stable transfection of specific shRNA. *Wild type* (native) or control (transfected with scrambled shRNA sequences) GBM CSCs retain similar levels of *in vitro* self-renewal activity, expression of neural stem cell markers, and both are highly tumorigenic when orthotopically grafted in immunocompromised mice. Moreover, when grown in FBS-containing medium, *wild type* and control GBM CSCs differentiate into cells resembling the population composing the bulk of tumors mass [46, 49, 71], expressing neuronal and glial markers, but losing the tumorigenic activity in mouse models, according to the hierarchical model of tumor development. In this study, we show that the reduction of the expression of PrP^C^ in GBM CSCs: (a) decreases cell proliferation rate; (b) dramatically reduces self-renewal (as evaluated by spherogenesis activity) and clonogenic activities; (c) promotes spontaneous cell differentiation, characterized by down-regulation of specific stem cell markers involved in pluripotency and self-renewal, and inducing morphological and phenotypical (i.e. expression of GFAP) features of mature astrocytes. More importantly, all these functional alterations determine the loss of the ability to develop tumors in mice, thus causing the differentiation of GBM tumor-initiating cells into a non-tumorigenic subpopulation. This evidence suggests that PrP^C^ is a major regulator of GBM stem cell activity. Importantly, in glioma, the number of GFAP-expressing cells was reported to be inversely correlated to the level of tumor anaplasia, likely representing the content of undifferentiated, stem-like cells [72]. Our data are in line with this observation since we show that CSCs do not express GFAP, and that the loss of stemness induced by PrP^C^ down-regulation is correlated with the induction of this astrocytic marker. Furthermore, we observed that the down-regulation of PrP^C^ also favors CSC differentiation toward endothelial-like cells. In fact, when induced to assembly into vessel-like structures by growing in selective medium PrP-KO cells showed higher efficiency than control cells, although also these cells were able to undergo tube formation.

PrP^C^ role in the regulation of cell stemness has been also described in different cell models, involving normal neural stem cells: Prodromidou *et al.* observed that PrP^C^ depletion reduces proliferation rate and secondary neurosphere formation of neuronal progenitors isolated from mouse subventricular zone [73]; Santos *et al.* demonstrated that neurospheres formation from fetal forebrain of *PRNP*^(0/0)^ mice was significantly reduced when compared with the *wild-type* counterparts [28]; Mohanty *et al*. showed a significant reduction in proliferation and clonogenic potential in human mesenchymal stem cells when PrP expression was down-regulated [74]. Similarly, PrP^C^ was also shown to influence the proliferation of human CSCs in which a population of CD44^+^/PrP^C+^ cells was isolated from primary colorectal tumors, showing an enhanced tumor-initiating and metastatic ability [37].

Our data extend this evidence to human GBM CSCs, reporting that the reduction of PrP^C^ expression is associated with a significant reduction of proliferation rate and tumorigenicity. We demonstrate that these effects are related to the decrease of the expression of different transcription factors, such as Sox2 and NANOG, essential for maintaining self-renewal or pluripotency of both normal stem cells and CSCs, resulting in the activation of a differentiating program and loss of tumorigenicity. Interestingly, these factors have been linked to abnormal proliferation and oncogenic transformation [75] and NANOG is up-regulated in radio-resistant GBM stem cells [76]. On the other hand, Sox2 down-regulation in GBM CSCs causes cell growth arrest and loss of in vivo tumorigenicity [77], further confirming the relevance of the alterations induced by PrP^C^-KO we highlight in this study.

The intracellular mechanisms responsible for these regulation have not been yet completely defined. It is known that PrP^C^, although mainly localized on the membrane, modulates different transduction mechanism including Fyn or other c-src-like kinases [30]. Thus, we can hypothesize that a constitutive activation of components of this kinase family by PrP^C^ might control the expression of stemness and self-renewal factors, as demonstrated for Lck in established glioma cell lines [78]. In fact, PrP^C^-dependent activation of c-src-like kinases (Fyn and Yes) sustains β-catenin activity, a main regulator of SOX2 expression [79]. Further studies are required to directly address this issue.

Different recent studies show that GBM CSCs play a central role in sustaining long-term tumor growth after chemotherapy [43, 80], underlying the importance of the identification of specific pharmacological targets in this GBM cell subpopulation. Other reports suggest that the induction of CSC differentiation could represent a new therapeutic strategy against GBM [81–83], and that tumors enriched in differentiated cells (i.e. co-expressing aldehyde dehydrogenase 1A1 and GFAP but not nestin or Sox2) correlate with better patients' survival [84]. Interestingly, PrP^C^ displays a significant plasticity in human tumors and thus it represents a potential therapeutic target. For example, PrP^C^ overexpression is induced in breast cancer cells by endoplasmic reticulum stress, conferring to tumor cells increased resistance to cytotoxic stimuli [85]. All in all, the induction of GBM CSC differentiation by PrP^C^ down-regulation, or its functional inhibition, could represent a relevant novel approach in this field.

In conclusion, our data point out that PrP^C^ plays a central role in GBM cell stemness and tumorigenesis, and it could represents a novel target for new pharmacological approaches.

## MATERIALS AND METHODS

### Antibodies and Reagents

Anti-PrP antibodies: 3F4 (mouse) (Signet Lab, London, UK), Saf 32 (mouse) (Bertin Pharma, France); anti-β actin (mouse), anti-GFAP (rabbit), anti-NANOG (rabbit) (Abcam, Cambridge, UK); anti-Sox2 (rabbit) (Merck Millipore, Darmstadt, Germany).

Secondary antibodies: Alexa-Fluor 488-conjugated anti-rabbit IgG (Molecular Probes, Invitrogen Corp., Carlsbad, CA, USA) for immunofluorescence, and horseradish peroxidase-linked anti-mouse or anti-rabbit IgG antiserum (GE Healthcare, Milano Italy) for Western blots.

### Human GBM specimens

Tumor specimens were obtained from Neurosurgery Dept. IRCCS-AOU San Martino-IST (Genova, Italy), after Institutional Ethical Committee approval of the informed consent provided to patients and the *ex vivo* human sample study. Tumors were diagnosed as primary WHO grade IV GBM. GBM1 (neural) occurred in a 67 year-old man, with a right hemisphere localization and a cortical development; GBM2 (neural) occurred in 48 year-old men, with a left hemisphere localization and a sub-cortical development; GBM3 (mesenchymal) occurred in 41 year-old women with a right fronto-temporal localization and cortical diffusion; GBM4 (neural) occurred in 70 year-old women with a right hemisphere localization and a sub-cortical development. Patients underwent first-time surgery and never received chemo- or radio-therapy. Immunohistochemical analysis showed a MIB index of 40% for GBM1 and GBM4, 60% for GBM2, and 35% for GBM3; all tumors were GFAP^+^ and no signs of meningeal invasion was detected, with the exception of GBM3. Upon arrival in the laboratory, tumor specimens were immediately processed to isolate single cells by mechanical dissociation and cell suspension was filtered through a 40 μm strainer (BD Biosciences, Buccinasco, MI, Italy) to remove aggregates, and cultured as previously described [46].

### Human GBM CSC cultures

Cells were grown in stem cell-permissive medium [DMEM-F12/Neurobasal (1:1), supplemented with 1% B27 (Life Technologies, California, USA), 2 mM L-glutamine (Lonza Srl, Milano, Italy), 100 u/ml penicillin/streptomycin (Lonza), 10 ng/ml bFGF and 20 ng/ml EGF (PeproTech, London, UK)] [47]. Cells were grown as monolayer on Matrigel (BD Biosciences Milano, Italy), as reported [46]. In these conditions, cells retain CSC features as routinely assessed by stem cell marker expression and *in vivo* tumorigenicity [46]. This culture condition was used to obtain easier evaluation of cell biology and biochemical experiments, rather than using non-adherent spheroids. To induce cell differentiation, CSC cultures were shifted to growth factor-deprived medium containing 10% fetal bovine serum (FBS, Lonza) for at least 2 wk. GBM CSC tumorigenicity was assayed by intracranial inoculation of 10^4^ cells/mouse, in 6–8-wk-old NOD/SCID mice (Charles River, Calco, Italy), in compliance with guidelines approved by Ethical Committee for animal use in cancer research at IRCCS-AOU San Martino-IST (Genova, Italy).

### Stable silencing of PrP expression in GBM CSC cultures

GBM CSCs were transfected with PrP shRNA Plasmid (h) (Santa Cruz Biotechnology, Santa Cruz, CA, USA); this is a pool of 3 target-specific lentiviral vector plasmids each encoding 19-25 nt (plus hairpin) shRNAs, designed to knock-down gene expression.

Briefly, 24 hours before the transfection, cells were seeded at 50-70% confluence in a six well tissue culture plate in stem cell permissive medium. Ten ml of shRNA plasmid DNA (1 μg), diluted in 90 ml of shRNA Plasmid Transfection Medium were mixed with a solution containing 4 μl shRNA Plasmid Transfection Reagent and 96 μl of shRNA Plasmid Transfection Medium (Santa Cruz Biotechnology) for 45 min according to the manufacturer's instructions. Cells were washed twice with 2 ml of shRNA Transfection Medium, and for each transfection, 0.8 ml shRNA Plasmid Transfection Medium and 200 μl shRNA Plasmid DNA/shRNA Plasmid Transfection Reagent was added. After 8 hrs at 37°C in a CO_2_ incubator, 1 ml of DMEM-F12/Neurobasal medium, containing 2 times the normal serum and antibiotics concentrations, was added. After additional 20 hrs, infected cells were moved in stem cell permissive medium and selected by adding puromycin (5 μg/ml). The same procedure was performed with control plasmid encoding a scrambled shRNA sequence that will not lead to the specific degradation of any known cellular mRNA.

Using this protocol we generate GBM-PrP-KO and GBM-Scr (control) cells.

### Western blot

Cells were lysed in a buffer containing 20 mM Tris-HCl pH 7.4, 140 mM NaCl, 2 mM EDTA, 2 mM EGTA, 10% glycerol, 1% NP-40, 1 mM dithiothreitol, 1 mM sodium orthovanadate, 1 mM phenylmethylsulphonyl fluoride, and the “Complete” protease inhibitor cocktail (Roche, Monza, Italy), as reported [86]. Twenty-five micrograms of proteins from each sample were size-fractionated by 12% SDS-PAGE, transferred to a poly-vinylidene difluoride membrane (Bio-Rad Laboratories, Hercules, CA, USA), and probed with the primary antibodies. The secondary antibody was a horseradish peroxidase-linked anti-rabbit IgG or anti-mouse IgG antiserum (GE Healthcare, Milano, Italy). Antibody-reactive bands have been detected by ECL (GE Healthcare) using ChemiDoc™ MP Systems (Bio-Rad, Segrate, Italy).

### Immunocytofluorescence

GBM cells were fixed with 4% paraformaldehyde, permeabilized with PBS/0.1% Triton X-100, blocked with normal goat serum and immunostained with anti-GFAP antibody (rabbit polyclonal, 1:1,0000, Abcam, Cambridge, UK). Fluorochrome-conjugated antibody (goat anti-rabbit Alexa Fluor-488, Molecular Probes, Oregon, USA) was added, nuclei were counterstained with DAPI (Sigma-Aldrich, Milano, Italy), and cell morphology evidenced by Dil (Molecular Probes) a lipophilic red-fluorescent dye that stains plasma-membranes and cytoplasmic vesicles [6]. Slides were photographed with DM2500 microscope (Leica, Milano, Italy) equipped with DFC350FX digital camera (Leica).

### Cell proliferation assay

Mitochondrial activity, an index of cell viability, was evaluated by measuring the reduction of 3-(4,5-dimethylthiazol-2-yl)-2,5-diphenyltetrazolium bromide (MTT, Sigma-Aldrich). After treatments, cells were incubated with MTT (2 mg/ml) for 1h, formazan crystals dissolved in DMSO and absorbance measured at 570 nm [87].

### Cell Counting

CSCs from different GBMs, grown in standard conditions for 3 days, were counted with an automated cell counter every 24 hours. Cells were harvested and the cell suspension diluted 1:10 in sterile PBS and mixed with an equal volume of 0.4 % Trypan Blue solution to evaluate the number of live/dead cells using TC20 Cell Counter (Bio-Rad Laboratories, Inc., Hercules, CA) [88].

### BrdU incorporation ELISA

DNA synthesis was evaluated by assessing 5-bromo-2′-deoxyuridine (BrdU) incorporation during DNA synthesis (Cell proliferation ELISA, Roche), as reported [71].

### Clonogenicity assay

To verify the effect of PrP^C^ down-regulation on GBM CSC stemness, an *in vitro* assay based on the ability of a single cell to grow into a colony was used [89]. GBM-Scr and GBM-PrP-KO cells were seeded with scalar dilution in 96-well microplates without Matrigel to reach a concentration lower than 1 cell/well. After 24 h, wells were visually inspected under a light microscope, and wells containing no live cells or more than 1 cell were excluded. Cells were maintained in complete medium for a week to allow the clonal growth. After 7 days clones were visually inspected by light microscopy and counted by three independent operators.

### Sphere-formation assay

We used the sphere limiting dilution analysis to evaluate sphere formation in GBM-Scr and GBM-PrP-KO. GBM cells were cultured as primary spheres. Morphology of the spheres developed in the wells was evaluated using a digital camera Leica ICC50 HD (Leica) mounted on a transmitted light microscope DM IL (Leica) to image each individual well [48]. Subsequently, spheres were dissociated with Accutase™ for 5 min at 37°C and mechanically disaggregated until a single cell suspension was achieved. Cells were then plated at densities ranging from 100 to 0.01 cells across two 96 well plates in 200μl of complete medium with 16 replicates for each dilution and then evaluated for secondary sphere formation after 15 days in culture. We scored each well for the absence or presence of sphere growth to determine the fraction of negative wells. The plot shows natural log transformation for the fraction of non-responding wells (y-axis) versus plating density (x-axis). The probability of forming a sphere is determined by the x intercept (cell density) when y=−1 [53].

### Endothelial tube formation assays

μ–Slide Angiogenesis (Ibidi, Munich, Germany) were coated with Matrigel (BD Biosciences) and allowed to polymerize at 37°C for 30 min. 10^4^ cells were subsequently seeded and incubated at 37°C for 24h, in Endogro medium to induce endothelial transdifferentiation (Merck Millipore, Darmstadt, Germany). Tube-like structures were photographed using a phase contrast microscope. To quantify the results, we counted the mean loop perimeter and the number of branch points, in which at least 3 tubes joined, using the ImageJ software.

### RNA extraction and quantitative real-time PCR (qRT-PCR)

Total RNA was extracted using the High Pure RNA Isolation Kit (Roche), according to the manufacturer's instruction, and reverse transcribed into cDNA using the iScript cDNA Synthesis Kit (Bio-Rad) [90].

Single stranded cDNA products were analyzed by real-time PCR using the SsoFastTM EvaGreen mix (Bio-Rad) on a CFX96 Touch real-time PCR (Bio-Rad). Cycling conditions were set at 94°C for 30 s, 60°C for 30 s and 72°C for 30 s, for 37 cycles.

Primer sequences were designed on the mature transcripts:

NANOG: forward: 5′-GTCCCAAAGGCAAACA ACCC-3′; reverse: 5′-TTGACCGGGACCTTGTCTTC-3′;

Sox2: forward: 5′-CAGGAGTTGTCAAGGCAGA GA-3′; reverse: 5′-GTCCTAGTCTTAAAGAGGCAG CA-3′;

PrP: forward: 5′-AGTGGAACAAGCCGAGTAA GC 3′; reverse:

5′-GTCACTGCCGAAATGTATGATG-3′

GAPDH: forward: 5′-ACCCACTCCTCCACCTT TGA-3′; reverse: 5′-CTGTTGCTGTAGCCAAATTCGT-3

28S: forward: 5′-CCCAGTGCTCTGAATGTC AA-3′; reverse: 5′-AGTGGGAATCTCGTTCATCC-3′.

Levels of target genes in each sample were normalized on the basis of GAPDH and 28S amplification and reported as relative values. All qRT-PCR runs included negative controls without mRNA templates and cDNA transcription to check reagents for contaminations.

### *In vivo* tumorigenicity experiments

For *in vivo* experiments all the institutional and national guidelines for the care and use of laboratory animals were followed and the experimental plan approved by the committee for ethical animal use at IRCCS AOU S. Martino-IST, Genova, Italy. The tumor initiating potential of Scr- or PrP-silenced GBM cells was evaluated by orthotopic transplantation into 8 weeks-old NOD/SCID mice, as already described [46]. We injected 10,000 cells, since we previously showed that it allowed a tumor take rate of 100% of the animals, while this value was decreased to 40% with 1,000 cells and less than 20% with 100 cells [49]. Briefly, 10,000 cells were stereotactically injected into the striatum of ketamine-anesthetized mice, at 3 mm of depth, and tumors allowed to develop. Animals (n=4 in the different experimental groups) were monitored daily for neurological signs and all were sacrificed when first signs of morbidity was detected in one of the experimental groups. Collected brains were cryopreserved and 10-mm cryostat (Leica Microsystems) sections were cut. Sections bearing tumors were identified by H&E.

### Statistical analysis

Unless specified, all experiments were replicated three times. Data are reported as means ± SEM. Statistical analysis (ANOVA) was performed using IBM-SPSS 9.0 software (IBM Italia, Milano, Italy). *p* ≤ 0.05 was considered statistically significant.
